# *rac*-2-Methyl­amino-1,2-diphenyl­ethanol

**DOI:** 10.1107/S1600536812002012

**Published:** 2012-01-21

**Authors:** Ahmed Bari, Abdulrahman M. Al-Obaid, Seik Weng Ng

**Affiliations:** a Department of Pharmaceutical Chemistry, College of Pharmacy, King Saud University, Riyadh 11451, Saudi Arabia; b Department of Chemistry, University of Malaya, 50603 Kuala Lumpur, Malaysia; c Chemistry Department, Faculty of Science, King Abdulaziz University, PO Box 80203 Jeddah, Saudi Arabia

## Abstract

The dihedral angle between the two phenyl rings in the title compound, C_15_H_17_NO, is 52.9 (1)°. In the crystal, the mol­ecules are connected by O—H⋯N hydrogen bonds into centrosymmetric dimers. The amino H atom is not involved in hydrogen bonding.

## Related literature

For the use of chiral 2-(2-methyl­amino)-1,2-diphenyl­ethan-1-ol in organic synthesis, see: Gamsey *et al.* (2004 ▶).
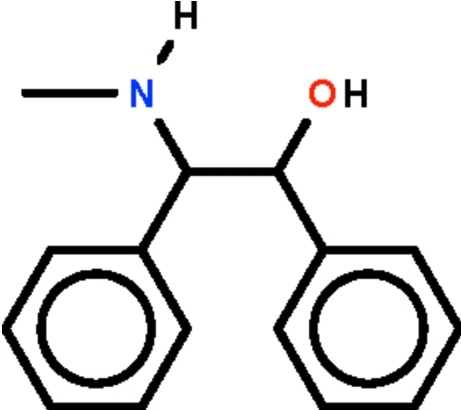


## Experimental

### 

#### Crystal data

C_15_H_17_NO
*M* *_r_* = 227.30
Monoclinic, 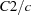

*a* = 27.4279 (7) Å
*b* = 5.69216 (11) Å
*c* = 17.1910 (5) Å
β = 116.223 (3)°
*V* = 2407.69 (10) Å^3^
*Z* = 8
Cu *K*α radiation
μ = 0.61 mm^−1^
*T* = 100 K
0.30 × 0.20 × 0.10 mm

#### Data collection

Agilent SuperNova Dual diffractometer with an Atlas detector
Absorption correction: multi-scan (*CrysAlis PRO*; Agilent, 2011 ▶) *T*_min_ = 0.838, *T*_max_ = 0.942
4176 measured reflections
2373 independent reflections
2197 reflections with *I* > 2σ(*I*)
*R*_int_ = 0.014

#### Refinement

*R*[*F* ^2^ > 2σ(*F* ^2^)] = 0.034
*wR*(*F* ^2^) = 0.092
*S* = 1.03
2373 reflections
164 parameters
H atoms treated by a mixture of independent and constrained refinement
Δρ_max_ = 0.29 e Å^−3^
Δρ_min_ = −0.22 e Å^−3^

### 

Data collection: *CrysAlis PRO* (Agilent, 2011 ▶); cell refinement: *CrysAlis PRO*; data reduction: *CrysAlis PRO*; program(s) used to solve structure: *SHELXS97* (Sheldrick, 2008 ▶); program(s) used to refine structure: *SHELXL97* (Sheldrick, 2008 ▶); molecular graphics: *X-SEED* (Barbour, 2001 ▶); software used to prepare material for publication: *publCIF* (Westrip, 2010 ▶).

## Supplementary Material

Crystal structure: contains datablock(s) global, I. DOI: 10.1107/S1600536812002012/bt5789sup1.cif

Structure factors: contains datablock(s) I. DOI: 10.1107/S1600536812002012/bt5789Isup2.hkl

Supplementary material file. DOI: 10.1107/S1600536812002012/bt5789Isup3.cml

Additional supplementary materials:  crystallographic information; 3D view; checkCIF report

## Figures and Tables

**Table 1:** Hydrogen-bond geometry (Å, °)

*D*—H⋯*A*	*D*—H	H⋯*A*	*D*⋯*A*	*D*—H⋯*A*
O1—H1o⋯N1^i^	0.92 (2)	1.88 (2)	2.799 (1)	172 (2)

Symmetry code: (i) 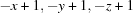.

**Fig. 1.**
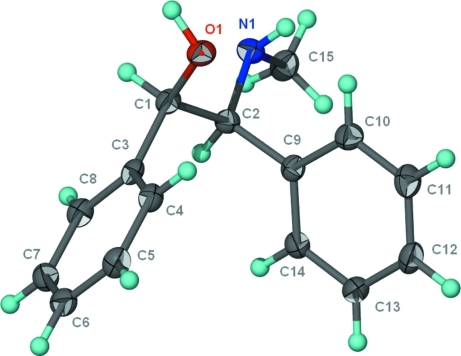
Thermal ellipsoid plot (Barbour, 2001) of C15H17NO at the 70% probability level; hydrogen atoms are drawn as spheres of arbitrary radius.

## supplementary crystallographic information

### Comment

Optically active 2-(2-methylamino)-1,2-diphenylethanol is used in asymmetric synthesis, *e.g.*, the asymmetric hydrogenation of chiral vinyloxazaboralidines (Gamsey *et al.*, 2004). The unsubstitued homolog is used for the palladium-assisted chiral tandem alkylation and carbonylative coupling reactions. The crystal structure of the *racemic* 2-(metahylamino)-1,2-diphenylethanol (Scheme I) is presented here.

The aromatic rings of the ethyl chain are staggered, the twist being 52.9 (1) °. The hydroxy group is hydrogen-bond donor to the amino group of an adjacent molecule; the amino group is hydrogen-bond donor to the hydroxy group of another molecule. The hydrogen bonds generate a linear chain running along [0 1 0] (Table 1).

### Experimental

The compound was obtained commercially, and crystals were grown from its solution in ethanol.

### Refinement

Carbon-bound H-atoms were placed in calculated positions [C–H 0.95 to 1.0 Å, *U*_iso_(H) 1.2 to 1.5*U*_eq_(C)] and were included in the refinement in the riding model approximation.

The amino and hydroxy H-atoms were located in a difference Fourier map, and were freely refined.

### Crystal data

**Table:**

C_15_H_17_NO	*F*(000) = 976
*M**_r_* = 227.30	*D*_x_ = 1.254 Mg m^−^^3^
Monoclinic, *C*2/*c*	Cu *K*α radiation, λ = 1.54184 Å
Hall symbol: -C 2yc	Cell parameters from 2861 reflections
*a* = 27.4279 (7) Å	θ = 2.9–74.3°
*b* = 5.69216 (11) Å	µ = 0.61 mm^−^^1^
*c* = 17.1910 (5) Å	*T* = 100 K
β = 116.223 (3)°	Prism, colorless
*V* = 2407.69 (10) Å^3^	0.30 × 0.20 × 0.10 mm
*Z* = 8	

### Data collection

**Table:**

Agilent SuperNova Dual diffractometer with an Atlas detector	2373 independent reflections
Radiation source: SuperNova (Cu) X-ray Source	2197 reflections with *I* > 2σ(*I*)
Mirror	*R*_int_ = 0.014
Detector resolution: 10.4041 pixels mm^-1^	θ_max_ = 74.5°, θ_min_ = 3.6°
ω scan	*h* = −26→34
Absorption correction: multi-scan (CrysAlis PRO; Agilent, 2011)	*k* = −6→4
*T*_min_ = 0.838, *T*_max_ = 0.942	*l* = −19→21
4176 measured reflections	

### Refinement

**Table:**

Refinement on *F*^2^	Secondary atom site location: difference Fourier map
Least-squares matrix: full	Hydrogen site location: inferred from neighbouring sites
*R*[*F*^2^ > 2σ(*F*^2^)] = 0.034	H atoms treated by a mixture of independent and constrained refinement
*wR*(*F*^2^) = 0.092	*w* = 1/[σ^2^(*F*_o_^2^) + (0.0476*P*)^2^ + 1.6566*P*] where *P* = (*F*_o_^2^ + 2*F*_c_^2^)/3
*S* = 1.03	(Δ/σ)_max_ = 0.001
2373 reflections	Δρ_max_ = 0.29 e Å^−^^3^
164 parameters	Δρ_min_ = −0.22 e Å^−^^3^
0 restraints	Extinction correction: *SHELXL97* (Sheldrick, 2008), Fc^*^=kFc[1+0.001xFc^2^λ^3^/sin(2θ)]^-1/4^
Primary atom site location: structure-invariant direct methods	Extinction coefficient: 0.0042 (2)

### Special details

**Table:**

Geometry. All e.s.d.'s (except the e.s.d. in the dihedral angle between two l.s. planes) are estimated using the full covariance matrix. The cell e.s.d.'s are taken into account individually in the estimation of e.s.d.'s in distances, angles and torsion angles; correlations between e.s.d.'s in cell parameters are only used when they are defined by crystal symmetry. An approximate (isotropic) treatment of cell e.s.d.'s is used for estimating e.s.d.'s involving l.s. planes.

### Fractional atomic coordinates and isotropic or equivalent isotropic displacement parameters (Å^2^)

**Table:**

	*x*	*y*	*z*	*U*_iso_*/*U*_eq_	
O1	0.56414 (3)	0.35675 (13)	0.57996 (5)	0.0187 (2)	
N1	0.49951 (4)	0.73117 (17)	0.59666 (6)	0.0174 (2)	
C1	0.57758 (4)	0.59658 (19)	0.57561 (7)	0.0159 (2)	
H1	0.5564	0.6491	0.5142	0.019*	
C2	0.55920 (4)	0.74825 (18)	0.63208 (7)	0.0152 (2)	
H2	0.5679	0.9151	0.6250	0.018*	
C3	0.63724 (4)	0.63255 (19)	0.59947 (6)	0.0157 (2)	
C4	0.67644 (4)	0.46581 (19)	0.64578 (7)	0.0178 (2)	
H4	0.6660	0.3244	0.6637	0.021*	
C5	0.73091 (5)	0.5046 (2)	0.66609 (7)	0.0201 (3)	
H5	0.7574	0.3897	0.6977	0.024*	
C6	0.74656 (4)	0.7110 (2)	0.64018 (7)	0.0198 (3)	
H6	0.7836	0.7365	0.6535	0.024*	
C7	0.70781 (5)	0.8798 (2)	0.59482 (7)	0.0196 (3)	
H7	0.7184	1.0217	0.5774	0.023*	
C8	0.65356 (4)	0.84107 (19)	0.57486 (7)	0.0183 (2)	
H8	0.6272	0.9575	0.5441	0.022*	
C9	0.58952 (4)	0.69014 (19)	0.72835 (7)	0.0156 (2)	
C10	0.57931 (5)	0.4847 (2)	0.76303 (7)	0.0203 (3)	
H10	0.5521	0.3788	0.7263	0.024*	
C11	0.60851 (5)	0.4332 (2)	0.85085 (7)	0.0220 (3)	
H11	0.6011	0.2927	0.8735	0.026*	
C12	0.64843 (5)	0.5860 (2)	0.90549 (7)	0.0215 (3)	
H12	0.6685	0.5504	0.9654	0.026*	
C13	0.65873 (4)	0.7908 (2)	0.87190 (7)	0.0217 (3)	
H13	0.6859	0.8965	0.9089	0.026*	
C14	0.62935 (4)	0.8422 (2)	0.78388 (7)	0.0184 (2)	
H14	0.6367	0.9834	0.7615	0.022*	
C15	0.47651 (5)	0.9274 (2)	0.62451 (7)	0.0219 (3)	
H15A	0.4381	0.8962	0.6083	0.033*	
H15B	0.4798	1.0722	0.5963	0.033*	
H15C	0.4963	0.9453	0.6876	0.033*	
H1O	0.5420 (7)	0.316 (3)	0.5233 (12)	0.047 (5)*	
H1N	0.4909 (6)	0.597 (3)	0.6155 (9)	0.027 (4)*	

### Atomic displacement parameters (Å^2^)

**Table:**

	*U*^11^	*U*^22^	*U*^33^	*U*^12^	*U*^13^	*U*^23^
O1	0.0215 (4)	0.0148 (4)	0.0180 (4)	−0.0025 (3)	0.0070 (3)	−0.0017 (3)
N1	0.0150 (4)	0.0174 (5)	0.0190 (5)	−0.0005 (3)	0.0067 (4)	0.0003 (4)
C1	0.0181 (5)	0.0147 (5)	0.0137 (5)	−0.0001 (4)	0.0059 (4)	0.0001 (4)
C2	0.0149 (5)	0.0147 (5)	0.0153 (5)	−0.0006 (4)	0.0060 (4)	−0.0001 (4)
C3	0.0189 (5)	0.0170 (5)	0.0118 (5)	0.0000 (4)	0.0073 (4)	−0.0027 (4)
C4	0.0211 (5)	0.0176 (5)	0.0160 (5)	0.0003 (4)	0.0094 (4)	−0.0001 (4)
C5	0.0199 (5)	0.0219 (6)	0.0183 (5)	0.0044 (4)	0.0084 (4)	0.0010 (4)
C6	0.0185 (5)	0.0239 (6)	0.0184 (5)	−0.0002 (4)	0.0094 (4)	−0.0029 (4)
C7	0.0229 (6)	0.0186 (5)	0.0193 (5)	−0.0027 (4)	0.0112 (5)	−0.0013 (4)
C8	0.0207 (5)	0.0178 (5)	0.0161 (5)	0.0018 (4)	0.0077 (4)	0.0006 (4)
C9	0.0161 (5)	0.0168 (5)	0.0153 (5)	0.0020 (4)	0.0082 (4)	−0.0008 (4)
C10	0.0233 (6)	0.0179 (6)	0.0188 (5)	−0.0033 (4)	0.0085 (5)	−0.0019 (4)
C11	0.0283 (6)	0.0191 (6)	0.0210 (6)	0.0010 (5)	0.0131 (5)	0.0032 (4)
C12	0.0219 (5)	0.0283 (6)	0.0151 (5)	0.0034 (5)	0.0088 (4)	0.0025 (4)
C13	0.0192 (5)	0.0290 (6)	0.0164 (5)	−0.0050 (5)	0.0075 (5)	−0.0029 (5)
C14	0.0195 (5)	0.0193 (5)	0.0182 (5)	−0.0025 (4)	0.0098 (4)	−0.0004 (4)
C15	0.0203 (5)	0.0264 (6)	0.0196 (6)	0.0037 (4)	0.0093 (5)	−0.0008 (4)

### Geometric parameters (Å, °)

**Table:**

O1—C1	1.4246 (13)	C7—C8	1.3888 (15)
O1—H1o	0.922 (18)	C7—H7	0.9500
N1—C15	1.4636 (14)	C8—H8	0.9500
N1—C2	1.4764 (13)	C9—C14	1.3900 (15)
N1—H1n	0.899 (15)	C9—C10	1.3961 (15)
C1—C3	1.5147 (14)	C10—C11	1.3922 (16)
C1—C2	1.5409 (14)	C10—H10	0.9500
C1—H1	1.0000	C11—C12	1.3889 (17)
C2—C9	1.5242 (14)	C11—H11	0.9500
C2—H2	1.0000	C12—C13	1.3842 (16)
C3—C4	1.3907 (15)	C12—H12	0.9500
C3—C8	1.3985 (15)	C13—C14	1.3953 (15)
C4—C5	1.3934 (15)	C13—H13	0.9500
C4—H4	0.9500	C14—H14	0.9500
C5—C6	1.3900 (16)	C15—H15A	0.9800
C5—H5	0.9500	C15—H15B	0.9800
C6—C7	1.3887 (16)	C15—H15C	0.9800
C6—H6	0.9500		
			
C1—O1—H1O	104.6 (11)	C6—C7—H7	120.0
C15—N1—C2	111.99 (9)	C8—C7—H7	120.0
C15—N1—H1N	107.9 (9)	C7—C8—C3	120.75 (10)
C2—N1—H1N	109.2 (9)	C7—C8—H8	119.6
O1—C1—C3	112.89 (8)	C3—C8—H8	119.6
O1—C1—C2	109.85 (8)	C14—C9—C10	118.25 (10)
C3—C1—C2	111.63 (8)	C14—C9—C2	119.79 (9)
O1—C1—H1	107.4	C10—C9—C2	121.95 (10)
C3—C1—H1	107.4	C11—C10—C9	120.78 (10)
C2—C1—H1	107.4	C11—C10—H10	119.6
N1—C2—C9	114.03 (8)	C9—C10—H10	119.6
N1—C2—C1	108.37 (8)	C12—C11—C10	120.36 (11)
C9—C2—C1	112.89 (8)	C12—C11—H11	119.8
N1—C2—H2	107.1	C10—C11—H11	119.8
C9—C2—H2	107.1	C13—C12—C11	119.37 (10)
C1—C2—H2	107.1	C13—C12—H12	120.3
C4—C3—C8	118.85 (10)	C11—C12—H12	120.3
C4—C3—C1	122.15 (10)	C12—C13—C14	120.16 (11)
C8—C3—C1	119.00 (9)	C12—C13—H13	119.9
C3—C4—C5	120.48 (10)	C14—C13—H13	119.9
C3—C4—H4	119.8	C13—C14—C9	121.09 (10)
C5—C4—H4	119.8	C13—C14—H14	119.5
C6—C5—C4	120.17 (10)	C9—C14—H14	119.5
C6—C5—H5	119.9	N1—C15—H15A	109.5
C4—C5—H5	119.9	N1—C15—H15B	109.5
C7—C6—C5	119.77 (10)	H15A—C15—H15B	109.5
C7—C6—H6	120.1	N1—C15—H15C	109.5
C5—C6—H6	120.1	H15A—C15—H15C	109.5
C6—C7—C8	119.97 (10)	H15B—C15—H15C	109.5
			
C15—N1—C2—C9	73.91 (11)	C6—C7—C8—C3	−0.40 (16)
C15—N1—C2—C1	−159.45 (9)	C4—C3—C8—C7	1.16 (15)
O1—C1—C2—N1	−62.37 (10)	C1—C3—C8—C7	−179.41 (9)
C3—C1—C2—N1	171.61 (8)	N1—C2—C9—C14	−130.40 (10)
O1—C1—C2—C9	64.92 (11)	C1—C2—C9—C14	105.35 (11)
C3—C1—C2—C9	−61.09 (11)	N1—C2—C9—C10	50.82 (13)
O1—C1—C3—C4	−18.78 (14)	C1—C2—C9—C10	−73.43 (12)
C2—C1—C3—C4	105.54 (11)	C14—C9—C10—C11	−0.34 (16)
O1—C1—C3—C8	161.81 (9)	C2—C9—C10—C11	178.45 (10)
C2—C1—C3—C8	−73.87 (12)	C9—C10—C11—C12	−0.04 (17)
C8—C3—C4—C5	−0.97 (15)	C10—C11—C12—C13	0.32 (17)
C1—C3—C4—C5	179.62 (10)	C11—C12—C13—C14	−0.23 (17)
C3—C4—C5—C6	0.04 (16)	C12—C13—C14—C9	−0.16 (17)
C4—C5—C6—C7	0.74 (16)	C10—C9—C14—C13	0.44 (16)
C5—C6—C7—C8	−0.55 (16)	C2—C9—C14—C13	−178.38 (10)

### Hydrogen-bond geometry (Å, °)

**Table:**

*D*—H···*A*	*D*—H	H···*A*	*D*···*A*	*D*—H···*A*
O1—H1o···N1^i^	0.92 (2)	1.88 (2)	2.799 (1)	172 (2)

Symmetry codes: (i) −*x*+1, −*y*+1, −*z*+1.
